# Multidisciplinary Approach of Management for Obstetric Patient With Moderate to Severe Bicuspid Aortic Valve Stenosis: A Case Report

**DOI:** 10.7759/cureus.107629

**Published:** 2026-04-24

**Authors:** Farah AlShammari, Abdulrady Ibrahim, Fatemah Qasem

**Affiliations:** 1 Anesthesiology, Kuwait Institute for Medical Specializations, Kuwait City, KWT; 2 Anesthesiology and Critical Care, Maternity Hospital, Kuwait City, KWT

**Keywords:** cesearen section, critical aortic stenosis, multi-disciplinary team, obstetric anesthesi, va-ecmo

## Abstract

Bicuspid aortic valve stenosis presents unique challenges during pregnancy due to increased hemodynamic stress and the potential for cardiovascular collapse. Therefore, a multidisciplinary approach is essential to optimize maternal and neonatal outcomes. This case report explores the complex management of a pregnant patient with bicuspid aortic valve stenosis who declined termination.

A 20-year-old female at 17 weeks of gestation presented with progressive shortness of breath on exertion and fatigue. Serial transthoracic echocardiography (TTE) revealed a mean aortic valve gradient of 46 mmHg and an aortic valve area of 0.9 cm², indicating severe aortic stenosis. At 35 weeks of gestation, the patient developed syncope and shortness of breath at rest with a metabolic equivalents (METS) score <4, requiring hospital admission. At 37 weeks, a planned cesarean section was performed, with extracorporeal membrane oxygenation (ECMO) cannulas inserted prior to induction as a standby. The anesthetic technique included invasive monitoring and induction with midazolam, lidocaine, fentanyl, etomidate, and rocuronium, with maintenance using remifentanil infusion and sevoflurane. The baby was delivered within five minutes in good health, and the patient was transferred to the ICU for close observation.

Pregnancy poses considerable risks for patients with severe aortic stenosis, as physiological changes can precipitate cardiac decompensation. Successful management relies on meticulous planning, individualized care, and interdisciplinary collaboration, with anesthetic strategies focused on maintaining maternal hemodynamic stability while ensuring fetal well-being. This case highlights the importance of a multidisciplinary approach and tailored anesthetic and perioperative management to achieve favorable maternal and neonatal outcomes in patients with severe aortic stenosis.

## Introduction

Bicuspid aortic valve stenosis is the most common cause of primary heart disease in the obstetric population, accounting for 2% of total women of childbearing age [1]. Pregnancy in women with bicuspid aortic valve stenosis presents unique challenges due to increased hemodynamic stress and physiological changes, most importantly, increased stroke volume in the presence of fixed cardiac output. This can lead to significant risk and complications such as heart failure, arrhythmias, aortic dissection, and even maternal mortality [2-4]. Maternal and fetal risk stratification for the obstetric population with congenital heart disease is well described by the modified World Health Organization (mWHO) classification [5]. 

Management in these patients requires a multidisciplinary approach to mitigate the risk of maternal morbidity and mortality, starting from structured preconception counseling to a planned delivery strategy. While asymptomatic patients with mild, moderate, and even severe aortic stenosis can continue pregnancy to term, symptomatic patients with severe aortic stenosis or asymptomatic patients with impaired left ventricular function carry a significant risk of cardiac complications [6]. Proper patient education and counseling regarding aortic valve replacement are essential before pregnancy. Alternative management options may also be considered, including less invasive approaches such as percutaneous aortic valvuloplasty, as well as more invasive interventions like aortic valve replacement performed with normothermic cardiopulmonary bypass [6,7]. Moreover, perioperative anesthetic management is highly challenging and may result in serious complications.

A systematic approach is essential, with meticulous vigilance, close patient monitoring, and careful attention to detail while maintaining stable hemodynamics throughout the perioperative period [8,9]. This case report aims to emphasize the importance of a systemic approach to parturient patients with severe symptomatic aortic stenosis that should start as early as possible to ensure patient safety and mitigate the possible complications, and to emphasize the challenging anesthesia management and the use of extracorporeal membrane oxygenation (ECMO) as a standby strategy. 

## Case presentation

A 20-year-old female, gravida 2, para 1, at 17 weeks of gestation, presented with shortness of breath, episodes of syncope, and easy fatigability for one month (grade III according to the mWHO classification). She had a known history of congenital bicuspid aortic valve stenosis, diagnosed earlier in her life. During her previous pregnancy, she had milder symptoms, which presented near the end of her pregnancy term, compared to this current pregnancy. At that time, she underwent an uncomplicated cesarean section at 37 weeks of gestation under titered epidural anesthesia uneventfully. 

In her second pregnancy, the patient first presented to the anesthesia clinic at 17 weeks of gestation; she reported shortness of breath during physical exertion for one month, with limited functional capacity. Over time, her symptoms worsened, leading to shortness of breath even at rest and episodes of syncope and easy fatigability in her third trimester. During clinical examination, she looked fatigued and out of breath, with bilateral lower limb edema. Her heart rate was 90 beats per minute, sinus rhythm; her blood pressure was 110/60, and oxygen saturation was 95% in room air. A transthoracic echocardiogram was evident for significant progressive stenosis with a mean aortic valve gradient of 46 mmHg and an aortic valve area of 0.9 cm². She had a preserved left ventricular systolic function with an ejection fraction of 66%. Laboratory investigations at her initial outpatient visit and at 37 weeks of gestation are shown in Table 1.

**Table 1:** Laboratory results

Parameter	Initial results at 17 weeks of gestation	At 37 weeks of gestation	Normal range
Hemoglobin (Hb) (g/dl)	8.7	1.0	11.0-2.5
WBC (x 10^9 /L)	12	13.5	4.5-11.0
Platates (x 10^9/ L)	116	127	150-450
International normalized ratio (INR)	0.9	1.1	0.9-1.3
Activated partial thromboplastin time (aPPT) (seconds)	40	43	25-35
N-terminal pro b-type natriuretic peptide (NT- proBNP) (pg/ml)	280	70	< 100
Sodium (Na) (mmol/L)	130	137	135-145
Potassium (K) (mmol/L)	3.2	4	3.5-5.5
Creatinine (umol/L)	62	58	60-120
Blood urea nitrogen (BUN) (mmol/L)	7	8	10-20

A multidisciplinary team that includes obstetrics, cardiology, and anesthesia was established to ensure optimal patient care. Percutaneous valvuloplasty of the aortic valve was considered during her first presentation. The patient was admitted to the hospital and managed conservatively to alleviate symptoms of heart failure and discharged home with the following medications: bisoprolol and furosemide. Cardiology evaluation was planned every two weeks in her second trimester, then weekly in her third trimester. Then, an elective cesarean section was planned at 37 weeks to minimize the risk of exacerbating cardiac compromise during labor. 

She underwent a cesarean section under general anesthesia with standby ECMO support. Although titrated epidural anesthesia was used during her previous cesarean section, it was deemed suboptimal for the current delivery due to progression of aortic stenosis, worsening of her clinical condition, and the anticipation of hemodynamic instability. Anesthesia was initiated following the placement of invasive monitors, including an arterial line, central venous line, and insertion of venoarterial (VA) ECMO sheaths in the right femoral vein and artery. Hemodynamic goals were carefully monitored, given this moderate-to-severe aortic stenosis and her status as an obstetric patient: heart rate was in sinus rhythm and in the low normal range; blood pressure, systemic vascular resistance, and contractility were maintained; and pulmonary vascular resistance increase was prevented.

Throughout the procedure, the patient remained stable with minimal fluctuations in hemodynamics, and blood gas analysis was within the normal range. Heart rate was sinus and ranged between 60 and 75 beats per minute; invasive systolic blood pressure was between 100 and 120 mmHg, and diastolic blood pressure was 60 to 70 mmHg. Anesthesia was maintained using inhalational anesthetics, and a bispectral index (BIS) monitor was utilized to ensure the appropriate depth of anesthesia.

The baby was safely delivered and was in good health, followed by administration of 20 IU of oxytocin over four hours. At the end of the procedure, the patient was extubated smoothly and without complications. Postoperatively, the patient was admitted to the ICU for close observation. She remained stable and was discharged with appropriate follow-up plans. The patient was referred to the cardiology center to undergo aortic valve replacement surgery in the near future.

## Discussion

This case highlights the significant anesthetic challenges associated with managing severe bicuspid aortic valve stenosis during pregnancy, particularly in the setting of advanced gestation and symptomatic deterioration. Pregnancy induces substantial physiological changes, including increased blood volume, heart rate, and cardiac output, which may be poorly tolerated in patients with fixed cardiac output lesions such as severe aortic stenosis. These changes increase the risk of maternal cardiac decompensation, syncope, and even sudden cardiovascular collapse [10,11].

Current guidelines emphasize that severe symptomatic aortic stenosis in pregnancy is associated with high maternal and fetal risk, and careful multidisciplinary planning is essential for optimal outcomes [10]. Hemodynamic goals include maintaining adequate preload, avoiding tachycardia, preserving sinus rhythm, and preventing reductions in systemic vascular resistance [11,12]. In our case, the use of invasive monitoring and a carefully titrated anesthetic technique allowed for close hemodynamic control throughout the perioperative period.

The decision to proceed with general anesthesia was guided by the need for controlled airway management and hemodynamic stability, particularly given the patient’s worsening symptoms and limited functional capacity (metabolic equivalents (METS) score <4). The use of etomidate for induction minimized cardiovascular depression, while opioid-based anesthesia with remifentanil helped attenuate sympathetic responses [12]. Additionally, pre-induction placement of ECMO cannulas as a standby measure provided a safety net in anticipation of potential cardiovascular collapse, reflecting a proactive and high-risk management strategy described in similar critical cardiac cases [13].

Alternative approaches, such as regional anesthesia, may be considered in selected patients but carry the risk of sudden decreases in systemic vascular resistance, which can be poorly tolerated in severe aortic stenosis [10]. Similarly, percutaneous aortic valvuloplasty has been described as a bridge therapy during pregnancy; however, its use depends on patient stability, gestational age, and institutional expertise [14,15].

Neuraxial anesthesia in patients with severe aortic stenosis remains controversial and carries significant risk due to its potential to cause sudden and profound sympathetic blockade. This can lead to an acute reduction in systemic vascular resistance (SVR), resulting in decreased coronary perfusion pressure, compromised myocardial oxygen supply, and subsequent hemodynamic collapse. In fixed cardiac output states such as severe aortic stenosis, the heart is unable to compensate for these rapid changes, increasing the risk of severe hypotension, syncope, myocardial ischemia, and cardiac arrest.

Although carefully titrated neuraxial techniques (e.g., incremental epidural dosing) have been described in select cases, they require extreme caution, invasive monitoring, and immediate availability of vasopressor support. Even with these precautions, the unpredictable spread of local anesthetic and variable hemodynamic responses make this approach less favorable in symptomatic patients with advanced disease. Current literature generally supports avoiding single-shot spinal anesthesia in severe aortic stenosis due to the risk of abrupt sympathectomy. In contrast, general anesthesia allows for tighter hemodynamic control, controlled airway management, and the ability to rapidly respond to cardiovascular instability. This rationale supported our decision to avoid neuraxial anesthesia in this patient, given her symptomatic deterioration and limited functional capacity. These concerns are supported by existing evidence highlighting the hemodynamic instability associated with neuraxial techniques in fixed outflow obstruction lesions [8].

This case underscores the importance of individualized anesthetic planning, meticulous perioperative monitoring, and a multidisciplinary approach involving obstetrics, cardiology, cardiac surgery, and anesthesia teams. Early recognition of clinical deterioration and proactive planning are crucial in reducing maternal and fetal morbidity [10,12]. Limitations include the inherent nature of a single case report, which limits generalizability. Nevertheless, this case provides valuable insight into the management of high-risk cardiac conditions during pregnancy and highlights the importance of maintaining hemodynamic stability while ensuring fetal well-being.

## Conclusions

This case demonstrates that severe symptomatic bicuspid aortic valve stenosis during pregnancy represents a high-risk clinical scenario requiring meticulous, individualized management. Successful maternal and fetal outcomes depend on strict adherence to hemodynamic principles, such as maintaining preload, preserving sinus rhythm, avoiding tachycardia, and preventing reductions in systemic vascular resistance. A carefully planned general anesthetic approach, incorporating invasive monitoring and titrated induction with cardiovascularly stable agents, can provide optimal control in patients with limited functional reserve. The use of advanced strategies, such as pre-induction extracorporeal support standby, may offer an additional safety margin in critically ill patients at risk of sudden decompensation.

This report underscores the pivotal role of early multidisciplinary collaboration and proactive perioperative planning in managing complex cardiac disease during pregnancy. Although conclusions are limited by the single-case design, the experience reinforces that vigilant monitoring, timely intervention, and individualized anesthetic strategies are essential to minimize maternal and fetal morbidity and mortality.
